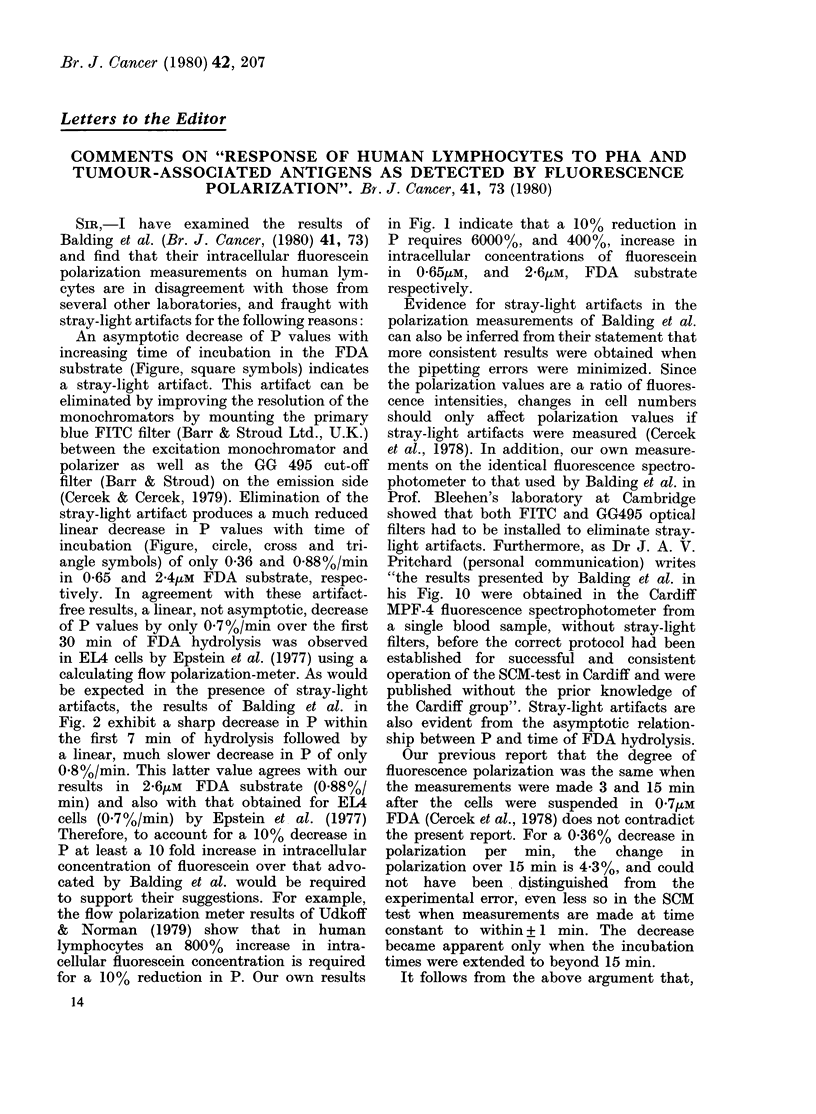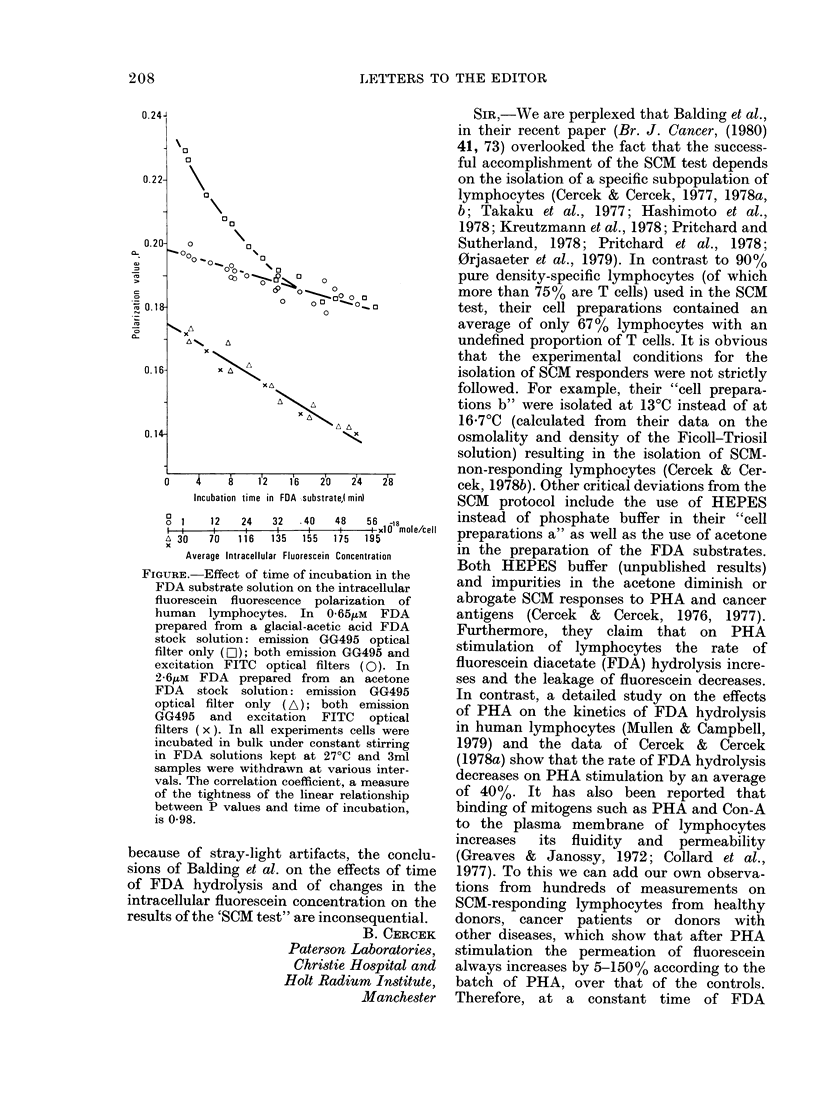# Comments on “Response of Human Lymphocytes to PHA and Tumour-Associated Antigens as Detected by Fluorescence Polarization”

**Published:** 1980-07

**Authors:** B. Cercek


*Br. J. Cancer* (1980) **42**, 207

### *Letters to the Editor*

### COMMENTS ON "RESPONSE OF HUMAN LYMPHOCYTES TO PHA AND TUMOUR-ASSOCIATED ANTIGENS AS DETECTED BY FLUORESCENCE POLARIZATION". *Br. J. Cancer*, 41, 73 (1980)

SIR,—I have examined the results of Balding *et al.* (*Br. J. Cancer*, (1980) **41,** 73) and find that their intracellular fluorescein polarization measurements on human lymcytes are in disagreement with those from several other laboratories, and fraught with stray-light artifacts for the following reasons:

An asymptotic decrease of P values with increasing time of incubation in the FDA substrate (Figure, square symbols) indicates a stray-light artifact. This artifact can be eliminated by improving the resolution of the monochromators by mounting the primary blue FITC filter (Barr & Stroud Ltd., U.K.) between the excitation monochromator and polarizer as well as the GG 495 cut-off filter (Barr & Stroud) on the emission side (Cercek & Cercek, 1979). Elimination of the stray-light artifact produces a much reduced linear decrease in P values with time of incubation (Figure, circle, cross and triangle symbols) of only 0·36 and 0·88%/min in 0·65 and 2·4$\mu$M FDA substrate, respectively. In agreement with these artifact-free results, a linear, not asymptotic, decrease of P values by only 0·7%/min over the first 30 min of FDA hydrolysis was observed in EL4 cells by Epstein *et al.* (1977) using a calculating flow polarization-meter. As would be expected in the presence of stray-light artifacts, the results of Balding *et al.* in Fig. 2 exhibit a sharp decrease in P within the first 7 min of hydrolysis followed by a linear, much slower decrease in P of only 0·8%/min. This latter value agrees with our results in 2·6$\mu$M FDA substrate (0·88%/min) and also with that obtained for EL4 cells (0·7%/min) by Epstein *et al.* (1977) Therefore, to account for a 10% decrease in P at least a 10 fold increase in intracellular concentration of fluorescein over that advocated by Balding *et al.* would be required to support their suggestions. For example, the flow polarization meter results of Udkoff & Norman (1979) show that in human lymphocytes an 800% increase in intracellular fluorescein concentration is required for a 10% reduction in P. Our own results in Fig. 1 indicate that a 10% reduction in P requires 6000%, and 400%, increase in intracellular concentrations of fluorescein in 0·65$\mu$M, and 2·6$\mu$M, FDA substrate respectively.

Evidence for stray-light artifacts in the polarization measurements of Balding *et al.* can also be inferred from their statement that more consistent results were obtained when the pipetting errors were minimized. Since the polarization values are a ratio of fluorescence intensities, changes in cell numbers should only affect polarization values if stray-light artifacts were measured (Cercek *et al.*, 1978). In addition, our own measurements on the identical fluorescence spectrophotometer to that used by Balding *et al.* in Prof. Bleehen's laboratory at Cambridge showed that both FITC and GG495 optical filters had to be installed to eliminate stray-light artifacts. Furthermore, as Dr J. A. V. Pritchard (personal communication) writes "the results presented by Balding *et al.* in his Fig. 10 were obtained in the Cardiff MPF-4 fluorescence spectrophotometer from a single blood sample, without stray-light filters, before the correct protocol had been established for successful and consistent operation of the SCM-test in Cardiff and were published without the prior knowledge of the Cardiff group". Stray-light artifacts are also evident from the asymptotic relationship between P and time of FDA hydrolysis.

Our previous report that the degree of fluorescence polarization was the same when the measurements were made 3 and 15 min after the cells were suspended in 0·7$\mu$M FDA (Cercek *et al.*, 1978) does not contradict the present report. For a 0·36% decrease in polarization per min, the change in polarization over 15 min is 4·3%, and could not have been distinguished from the experimental error, even less so in the SCM test when measurements are made at time constant to within $\pm 1$ min. The decrease became apparent only when the incubation times were extended to beyond 15 min.

It follows from the above argument that,

FIGURE.—Effect of time of incubation in the FDA substrate solution on the intracellular fluorescein fluorescence polarization of human lymphocytes. In 0·65$\mu$M FDA prepared from a glacial-acetic acid FDA stock solution: emission GG495 optical filter only (□); both emission GG495 and excitation FITC optical filters (○). In 2·6$\mu$M FDA prepared from an acetone FDA stock solution: emission GG495 optical filter only (△); both emission GG495 and excitation FITC optical filters (×). In all experiments cells were incubated in bulk under constant stirring in FDA solutions kept at 27°C and 3ml samples were withdrawn at various intervals. The correlation coefficient, a measure of the tightness of the linear relationship between P values and time of incubation, is 0·98.

because of stray-light artifacts, the conclusions of Balding *et al.* on the effects of time of FDA hydrolysis and of changes in the intracellular fluorescein concentration on the results of the 'SCM test" are inconsequential.

B. CERCEK
*Paterson Laboratories,*
*Christie Hospital and*
*Holt Radium Institute,*
*Manchester*